# QOI10/477: The medCERTAIN Project: Rating and certification of Internet health information using medPICS

**DOI:** 10.2196/jmir.1.suppl1.e103

**Published:** 1999-09-19

**Authors:** G Eysenbach, TL Diepgen, K Lampe, Ch Brickley

**Affiliations:** ^1^Institute for Clinical Social Medicine, HeidelbergGermany

**Keywords:** Quality of Internet Information, Rating, Filtering, PICS, RDF, XML

## Abstract

**Introduction:**

Health information on the Internet undergoes no quality control at the stage of production, thus its quality is highly variable, making it difficult for consumers to weed out the helpful from the harmful. In 1997, a uniform, machine readable vocabulary based on the WWW-Consortiums PICS-Standard (Platform for Internet Content Selection) was proposed and has become known as "medPICS". The advantage of this system is that it allows authors and third parties to label medical information on the Web using metadata which is standardized (PICS/RDF/XML), can be transmitted in a secure way, can be assigned by third parties and is machine-readable allowing the user being able to filter the information he wants. This presentation introduces a forthcoming European project dubbed Med-CERTAIN (MedPICS Certification and Rating of Trustful and Assessed Health Information on the Net), proposed by a consortium of partners from several European countries who are involved in the medical, Internet cataloguing and Web standardisation communities in co-operation with the W3C metadata interest group. The aim of this EU project is to provide a system which will allow European citizens to place greater trust in networked information in the domain of health information.

**Methods:**

Med-CERTAIN establishes a fully functional demonstrator for a self- and third-party rating system enabling patients and consumers to filter harmful health information and to positively identify and select high quality information. A standard rating vocabulary and Internet standards such as PICS/RDF/XML will be used to assign evaluative and descriptive meta-information to health information on the web. A trusted community of medically qualified volunteers will rate Internet information as they surf using advanced rating techniques such as Netscape's "Whats related" feature, bookmarklets (=Javascript-Code stored as Bookmarks), bookmark-uploads and JAVA tools.

**Results:**

The rating project will be starting in the year 2000. We are presently building up a database of several hundred medical volunteers who will rate the web and solicit institutional partners for this collaborative rating approach.

**Discussion:**

The project will demonstrate how PICS-based content rating and filtering technologies can automate and exploit value-adding resource description services. The proposed technology strategy combines a pragmatic use of simple existing technologies for data acquisition with a future-oriented standards policy intended to lead rather than follow the evolution of definitions for information-mediation services.

